# BNIP-2 retards breast cancer cell migration by coupling microtubule-mediated GEF-H1 and RhoA activation

**DOI:** 10.1126/sciadv.aaz1534

**Published:** 2020-07-31

**Authors:** Meng Pan, Ti Weng Chew, Darren Chen Pei Wong, Jingwei Xiao, Hui Ting Ong, Jasmine Fei Li Chin, Boon Chuan Low

**Affiliations:** 1 Mechanobiology Institute, 5A Engineering Drive 1, National University of Singapore, Singapore 117411, Singapore.; 2 Department of Biological Sciences, 14 Science Drive 4, National University of Singapore, Singapore 117543, Singapore.; 3 University Scholars Programme, 18 College Avenue East, Singapore 138593, Singapore.

## Abstract

Microtubules display dynamic turnover during cell migration, leading to cell contractility and focal adhesion maturation regulated by Rho guanosine triphosphatase activity. This interplay between microtubules and actomyosin is mediated by guanine nucleotide exchange factor (GEF)–H1 released after microtubule depolymerization or microtubule disconnection from focal adhesions. However, how GEF-H1 activates Rho upon microtubule disassembly remains elusive. Here, we found that BNIP-2, a BCH domain–containing protein that binds both RhoA and GEF-H1 and traffics with kinesin-1 on microtubules, is important for GEF-H1–driven RhoA activation upon microtubule disassembly. Depletion of BNIP-2 in MDA-MB-231 breast cancer cells decreases RhoA activity and promotes cell migration. Upon nocodazole-induced microtubule disassembly, the interaction between BNIP-2 and GEF-H1 increases, while knockdown of BNIP-2 reduces RhoA activation and cell rounding via uncoupling RhoA-GEF-H1 interaction. Together, these findings revealed that BNIP-2 couples microtubules and focal adhesions via scaffolding GEF-H1 and RhoA, fine-tuning RhoA activity and cell migration.

## Teaser

BNIP-2 acts as a brake on cell migration by scaffolding Rho and GEFs.

## INTRODUCTION

Directional cell migration, an important step of cancer invasion and metastasis, requires dynamic changes of the cytoskeleton and cell-matrix adhesions, which are tightly regulated by Rho guanosine triphosphatases (GTPases; e.g., RhoA, Rac1, and Cdc42) (*1*). Activations of RhoA and downstream RhoA effectors, including mDia and ROCK, result in stress fiber formation, actin polymerization, and actomyosin-dependent contraction (*2*). Although promoting migration in some cases, RhoA activation can inhibit single-cell migration through highly stable focal adhesions (*3*, *4*) and inhibit collective cell migration through the strong actin cables at the leading edge (*5*). Moreover, loss-of-function mutations in RhoA have been revealed in various cancers (*6*), and RhoA inactivation can promote colorectal cancer growth and skin tumor formation (*7*, *8*). Those findings suggest that RhoA can function as a tumor suppressor.

The activity of Rho GTPases is promoted by guanine nucleotide exchange factors (GEFs) and inhibited by GTPase-activating proteins (GAPs) (*9*). During cell migration, microtubule depolymerization results in actomyosin contractility through the activation of RhoA. The key player for the interplay between microtubules and actomyosin contractility is GEF-H1 (ARHGEF2). GEF-H1 is a microtubule-binding RhoA-specific GEF that activates RhoA when it is released from microtubules (*10*, *11*). It plays a critical role in focal adhesion dynamics and mechanosensing for migrating cells (*12*). GEF-H1 knockdown in mammary gland cell NMuMG decreases cell stiffness response to force and increases cell migration and invasion (*13*). Although it is known that GEF-H1 is spatially inhibited by microtubules (*14*) and microtubule capture by adhesion through KANK (*15*), less is known about how GEF-H1 activates RhoA after being released from microtubules and whether any scaffold proteins are involved in the process.

Rho GTPases and their regulators can be tethered together via scaffold proteins to be in close proximity for the interaction (*16*). For example, IQGAP1 and BPGAP1 scaffold for Ras signaling (*17*, *18*). The specific localization of scaffold proteins facilitates complex formation of Rho GTPases and regulators and determines the choice of downstream effectors, thus ensuring Rho signaling specificity (*19*). However, regulation of RhoA and RhoGEFs by scaffold proteins has yet to be fully delineated.

A varied number of Rho scaffold proteins contain a BNIP-2 and Cdc42GAP homology (BCH) domain (*20*). BCH domain is a functional subclass of the lipid-binding CRAL_TRIO/Sec14p superfamily across different species (*21*). BCH domains from different scaffold proteins can interact with different small GTPases, GAPs, or GEFs and fine-tune RhoA activity in different cell lines (*22*–*24*). As one of the BCH domain–containing proteins, BNIP-2 was first identified as an interacting partner to Cdc42GAP (*25*), later shown to promote cell elongation and membrane protrusion (*26*). Those studies suggest that BNIP-2 may regulate cell migration through the Rho-scaffolding BCH domain. Furthermore, BNIP-2 interacts with kinesin-1 light chain (KLC-1) and can be transported on microtubules (*27*), similar to its brain-specific homolog BNIP-H (*28*). Therefore, it is of interest to investigate whether BNIP-2 can scaffold microtubule-regulated GEF-H1 and RhoA for RhoA-mediated cancer cell migration.

In this study, we demonstrate that BNIP-2 is a scaffold protein for RhoA signaling and reveal that BNIP-2 couples microtubule and actin dynamics through scaffolding GEF-H1 and RhoA, regulating GEF-H1–driven Rho activity, focal adhesion dynamics, and cell migration. These findings reveal a previously unidentified mechanism by which microtubule disassembly–released GEF-H1 activates RhoA and focal adhesion dynamics via BNIP-2 scaffold protein to control cell migration.

## RESULTS

### BNIP-2 specifically interacts with RhoA via BCH domain

**Fig. 1:**
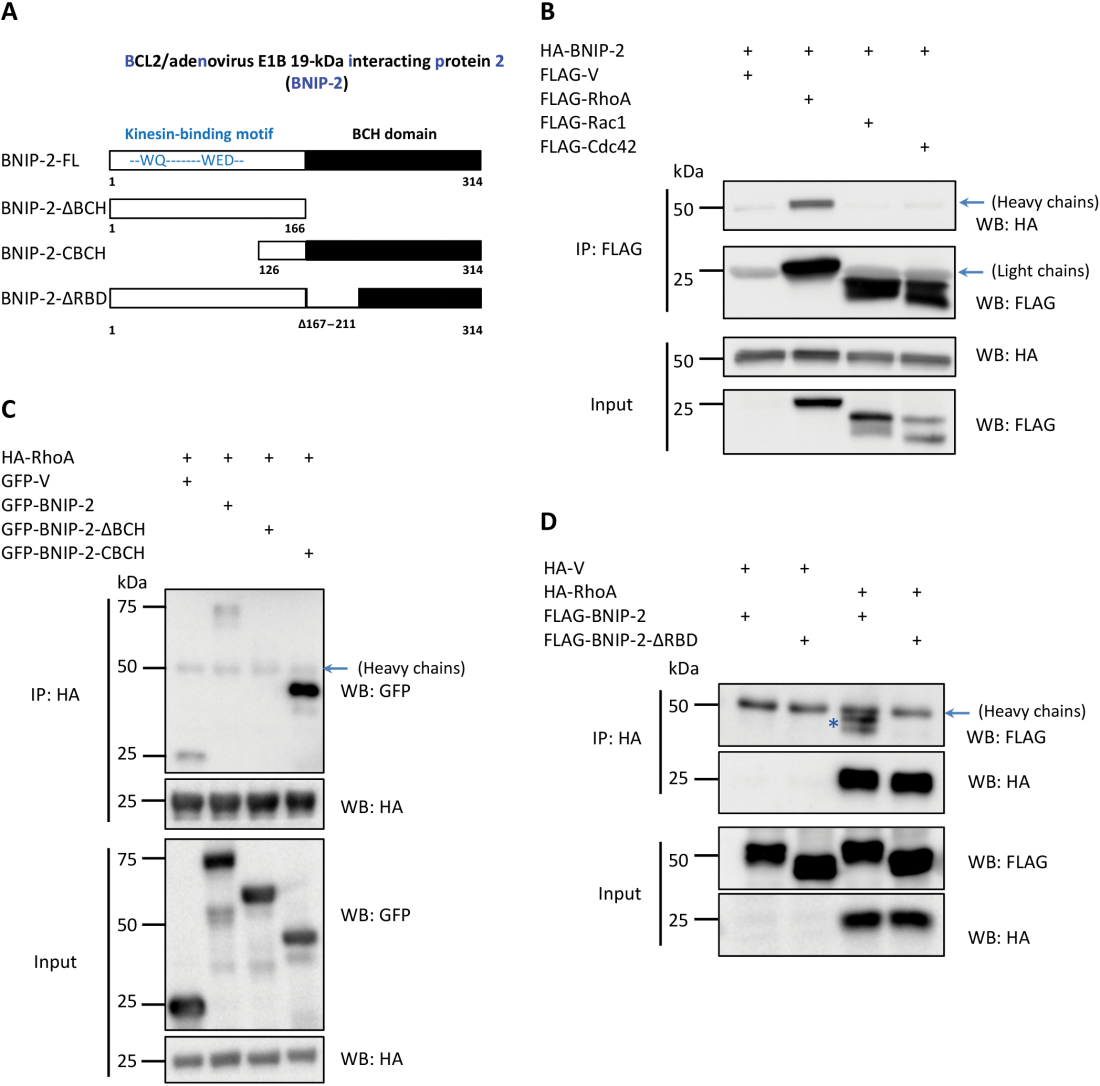
BNIP-2 interacts with RhoA via BCH domain. (**A**) Schematic representation of expression plasmids encoding BNIP-2-FL and its truncation and deletion mutants. In the BNIP-2-ΔRBD construct, 45 amino acids (167 to 211) are deleted. (**B**) BNIP-2 specifically binds to RhoA. Lysates of human embryonic kidney (HEK) 293T cells transiently expressing hemagglutinin (HA)–tagged BNIP-2 with FLAG-tagged empty vector, RhoA, Rac1, or Cdc42 were immunoprecipitated (IP) with anti-FLAG beads and then Western blotted (WB) with FLAG or HA antibodies. Blue arrows denote heavy chains and light chains from M2 beads that are present in all samples. (**C**) BNIP-2 uses its BCH domain to interact with RhoA. Lysates of HEK293T cells transiently expressing green fluorescent protein (GFP)–tagged BNIP-2, BNIP-2-ΔBCH, or BNIP-2-CBCH with HA-tagged RhoA were immunoprecipitated with anti-HA beads and then Western blotted with HA or GFP antibodies. Blue arrow denotes heavy chains from HA beads. (**D**) BNIP-2 uses the RBD-like region in the BCH domain to interact with RhoA. Lysates of HEK293T cells transiently expressing FLAG-tagged BNIP-2-FLand BNIP-2-ΔRBD with HA-tagged empty vector or RhoA were immunoprecipitated with anti-HA beads and then Western blotted with FLAG or HA antibodies. Blue arrow denotes heavy chains from HA beads that are present across all samples. BNIP-2 signal is shown just under the heavy chain in lane 3, denoted by the blue asterisk.

BNIP-2 has a BCH domain at the C terminus and a kinesin-binding motif at the N terminus (Fig. 1A). Some BCH domain–containing proteins, such as BNIP-XL and p50RhoGAP, have been reported to interact with RhoA (*23*, *24*). Therefore, we first investigated whether BNIP-2 could interact with RhoA or other Rho GTPases using coimmunoprecipitation assay. We showed that BNIP-2 could preferentially interact with RhoA over Rac1 and Cdc42 (Fig. 1B). The identification of the functional domain of BNIP-2 that binds to RhoA using coimmunoprecipitation revealed that both full-length BNIP-2 and BCH domain–containing BNIP-2-CBCH bind to RhoA, while BNIP-2 truncation without BCH domain (∆BCH) could not (Fig. 1C). Furthermore, a putative Rho-binding domain (RBD)–like region is mapped within BCH domain of BNIP-2, which is homologous to the RBD that was identified in BNIP-Sα by sequence analysis to various Rho-binding proteins (fig. S1) (*22*). The BNIP-2 mutant that lacks RBD region abolished the interaction with RhoA (Fig. 1D). These results show that BNIP-2 interacts with RhoA via the RBD region in BCH domain.

### BNIP-2 knockdown suppresses RhoA activity

**Fig. 2:**
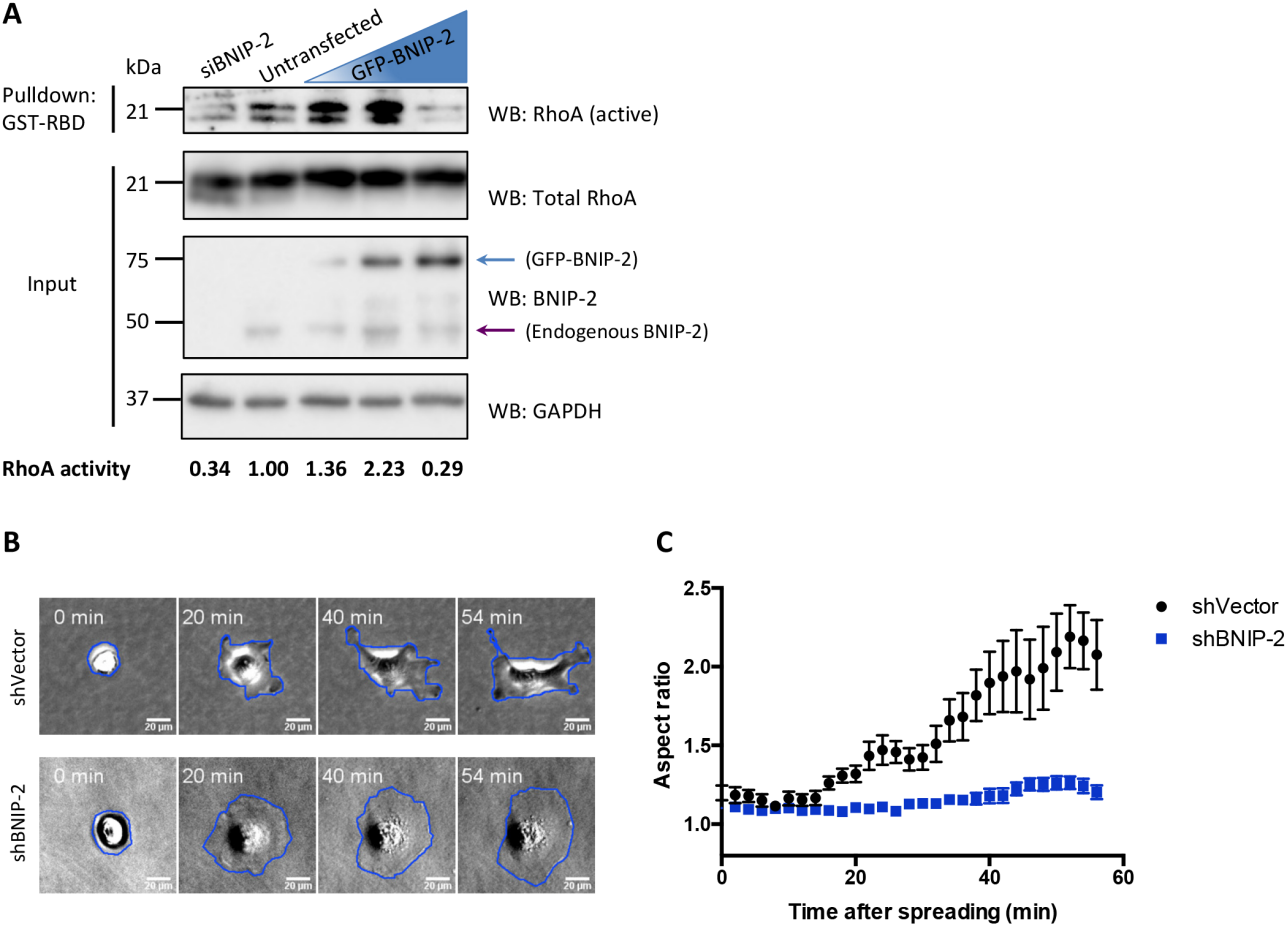
BNIP-2 knockdown suppresses RhoA activity and retards cell polarization during early cell spreading. (**A**) Endogenous RhoA activity of MDA-MB-231 cells was examined in the presence of different amounts of BNIP-2. Lysates of MDA-MB-231 cells transfected with BNIP-2–targeting siRNA (siBNIP-2) or transiently expressing gradually increasing amount of GFP-BNIP-2 (illustrated by blue triangle) were used for pulldown with immobilized glutathione *S*-transferase (GST)–RBD (GST-RBD) of rhotekin and then Western blotted with RhoA, BNIP-2, and glyceraldehyde-3-phosphate dehydrogenase (GAPDH) antibodies. Blue arrow denotes the GFP-BNIP-2, while purple arrow denotes the endogenous BNIP-2. The ratio of active RhoA to total RhoA is normalized to untransfected in lane 2 and labeled at the bottom. (**B**) Snapshots of shVector control and BNIP-2 knockdown cells during spreading on collagen I–coated plastic-bottom plates. Representative cells spreading at 0, 20, 40, and 54 min are displayed. (**C**) Cell aspect ratios are quantified and plotted for control and BNIP-2 knockdown cells during 60-min spreading. Data are means ± SEM of two independent experiments.

Earlier reports from our group showed that the binding of BCH domain to RhoA could either promote or inhibit RhoA activity, as evidenced by BNIP-XL, which interacts with RhoA using BCH domain and suppresses RhoA activity, and by p50RhoGAP, which sequesters RhoA via BCH domain to prevent RhoA activity being suppressed by its adjacent GAP domain (*23*, *24*). Hence, we examined whether BNIP-2 could promote or suppress RhoA activity in MDA-MB-231 cell, a highly migratory breast cancer cell line. RBD pulldown assay was performed to determine the active RhoA levels in MDA-MB-231 cells in the presence of endogenous BNIP-2, with small interfering RNA (siRNA)–induced knockdown of BNIP-2, as well as with different overexpression levels of green fluorescent protein (GFP)–BNIP-2 (Fig. 2A). The level of active RhoA in MDA-MB-231 cells was greatly reduced after depletion of BNIP-2 (Fig. 2A, lanes 1 and 2), increased in cells overexpressing small amounts of GFP-BNIP-2 (Fig. 2A, lanes 3 and 4) and, however, decreased to be lower than control in cells with higher expression of BNIP-2 (Fig. 2A, lane 5). A wider range of BNIP-2 concentrations also displayed a similar trend of active RhoA and phosphorylated myosin light chain, an indicator of Rho-activated contractility (fig. S2A). These results suggest that BNIP-2 acts as an upstream regulator of RhoA and that the role toward RhoA activity can be either promotive or suppressive depending on the relative amount of its expression. Such concentration-dependent molecular regulation is a typical biphasic scaffold effect, as similarly shown by studies on IQGAP1 and BPGAP1 (*17*, *29*). Our results indicate that BNIP-2 may act as a scaffold protein for RhoA and regulate cellular behaviors that are controlled by RhoA activity. Since ectopic expression of BNIP-2 has different effects on RhoA, depending on BNIP-2 concentration, we focused on BNIP-2 knockdown approach to study the function of BNIP-2 in the subsequent work.

### BNIP-2 knockdown retards cell polarization during cell spreading

To investigate the functional consequence of BNIP-2 on RhoA-mediated cell behaviors, we generated MDA-MB-231 cell line with stable BNIP-2 knockdown (fig. S2B). RhoA-induced actomyosin contractility is required for cell symmetry breaking and stress fiber formation during early cell spreading (*3*, *30*). For examination of cell morphology during early cell spreading, control and BNIP-2 knockdown cells were seeded on collagen-coated dishes for live-cell imaging during early spreading (movie S1). Snapshots of cell spreading showed that control cells were already polarized at 20 min after seeding (Fig. 2B). In contrast, BNIP-2 knockdown cells still displayed impaired cell polarization even after 60 min of seeding (Fig. 2B). The process of cell polarization is analyzed by quantifying the cell aspect ratio as a function of spreading time (Fig. 2C). We observed that the aspect ratio of control cells increases notably faster than BNIP-2 knockdown cells. These results show that BNIP-2 knockdown retards cell polarization during cell spreading, most likely because of the reduced Rho activity.

### BNIP-2 knockdown promotes breast cancer cell migration

**Fig. 3:**
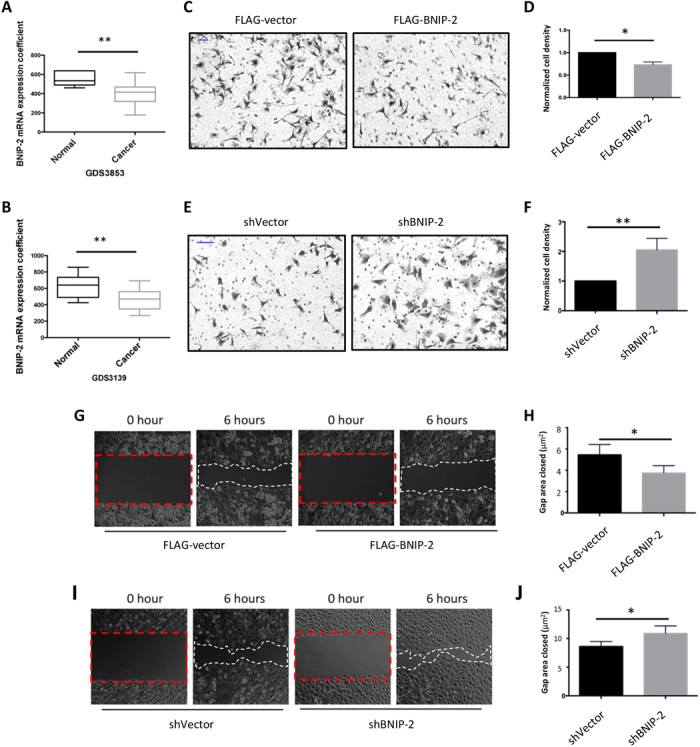
BNIP-2 knockdown promotes MDA-MB-231 cell migration. (**A** and **B**) BNIP-2 expression level is down-regulated in patient breast cancer samples in comparison to normal tissues. Microarray data were obtained from National Center for Biotechnology Information Gene Expression Omnibus database GDS3853 and GDS3139. Two-tailed *t* test was conducted. (A) In GDS3853, *P* < 0.01. (B) In GDS3139, *P* < 0.01. (**C** to **F**) BNIP-2 overexpression suppresses MDA-MB-231 cell transwell migration, while BNIP-2 knockdown promotes this process. (C) Representative images of transwell migration assay on MDA-MB-231 control and BNIP-2 overexpression cells. Transwell migration analysis was conducted using 10% fetal bovine serum–containing medium as a chemoattractant. Cells were fixed by 4% paraformaldehyde (PFA) after 6-hour migration. Cells migrated through and localized at the bottom side of the insert were stained with crystal violet for cell counting. (D) Statistical analysis between control and overexpression cells in transwell migration assay. Cell number per area was counted form randomly choosing sites and averaged. Final results presented here were normalized to the number of control cells (equals 1). Data are means ± SEM of four independent experiments, *P* < 0.05. (**E**) Representative images of transwell migration assay on MDA-MB-231 control and BNIP-2 knockdown cells. (**F**) Statistical analysis between control and knockdown cells in transwell migration assay. Data are means ± SEM of four independent experiments, *P* < 0.01. Scale bars in C and E, 100 μm. (**G** to **J**) BNIP-2 retards collective cell migration in MDA-MB-231 cell. (G) Stable BNIP-2–expressing MDA-MB-231 cells retard collective migration than control cells. (H) Statistical analysis for (G). Data are means ± SEM of five independent experiments, *P* < 0.05. (I) Knockdown of BNIP-2 increased the speed of collective cell migration. Red dashed rectangles denote gap area when stencile was removed (0 hour), and white dashed rectangles denote gap area after cells migrate collectively after 6 hours. (J) Statistical analysis for (I). Data are means ± SEM of four independent experiments, *P* < 0.05.

Since RhoA activity is essential for cancer cell migration and metastasis, we investigated the role of BNIP-2 in MDA-MB-231 cell migration. We found that the expression level of BNIP-2 is significantly reduced in breast tumor samples compared to normal tissues by analyzing two sets of breast carcinoma microarray data (GDS3853 and GDS3139; Fig. 3, A and B) and data from the TCGA (The Cancer Genome Atlas)–BRCA (Breast Invasive Carcinoma) database (fig. S3A). These results suggest that BNIP-2 could suppress cancer migration in vivo. Since BNIP-2 knockdown suppresses RhoA activity, we hypothesized that BNIP-2 knockdown could lead to increased motility of cancer cells through regulation of RhoA activity.

We examined whether BNIP-2 could suppress breast cancer cell migration using transwell migration assays. Cancer cell migration through transwell is reported to be inhibited by Rho activity (*3*, *4*). To verify that in our transwell assay, we treated MDA-MB-231 cells with Rho, ROCK, or myosin inhibitors and found that these treatments increase cell transwell migration (fig. S3B), suggesting that higher RhoA activity suppresses MDA-MB-231 cell migration. We then generated a stable MDA-MB-231 cell line that ectopically expresses FLAG-BNIP-2 (fig. S3C). Using transwell assay, we found that the stable BNIP-2–overexpressing cells showed decreased motility (Fig. 3, C and D). On the contrary, stable BNIP-2 knockdown MDA-MB-231 cells showed increased cell motility (Fig. 3, E and F). Furthermore, we found that cell motility increased by BNIP-2 knockdown can be rescued by transfection of hemagglutinin (HA)–BNIP-2 (fig. S3D). Similar to single-cell migration, collective cell migration is also suppressed by RhoA activity–induced actomyosin cables (*5*). Using wound healing assay, we showed that BNIP-2–overexpressing cells reduced migration (Fig. 3, G and H), while BNIP-2 depletion increased collective cell migration speed (Fig. 3, I and J), indicating that BNIP-2 also functions to suppress collective cell migration. Consistent with the information from breast tumor microarray datasets, our data showed that BNIP-2 depletion promotes cancer cell migration, while physiological level of BNIP-2 suppresses cell migration.

### BNIP-2 binds GEF-H1 and scaffolds for RhoA and GEF-H1 signaling

**Fig. 4:**
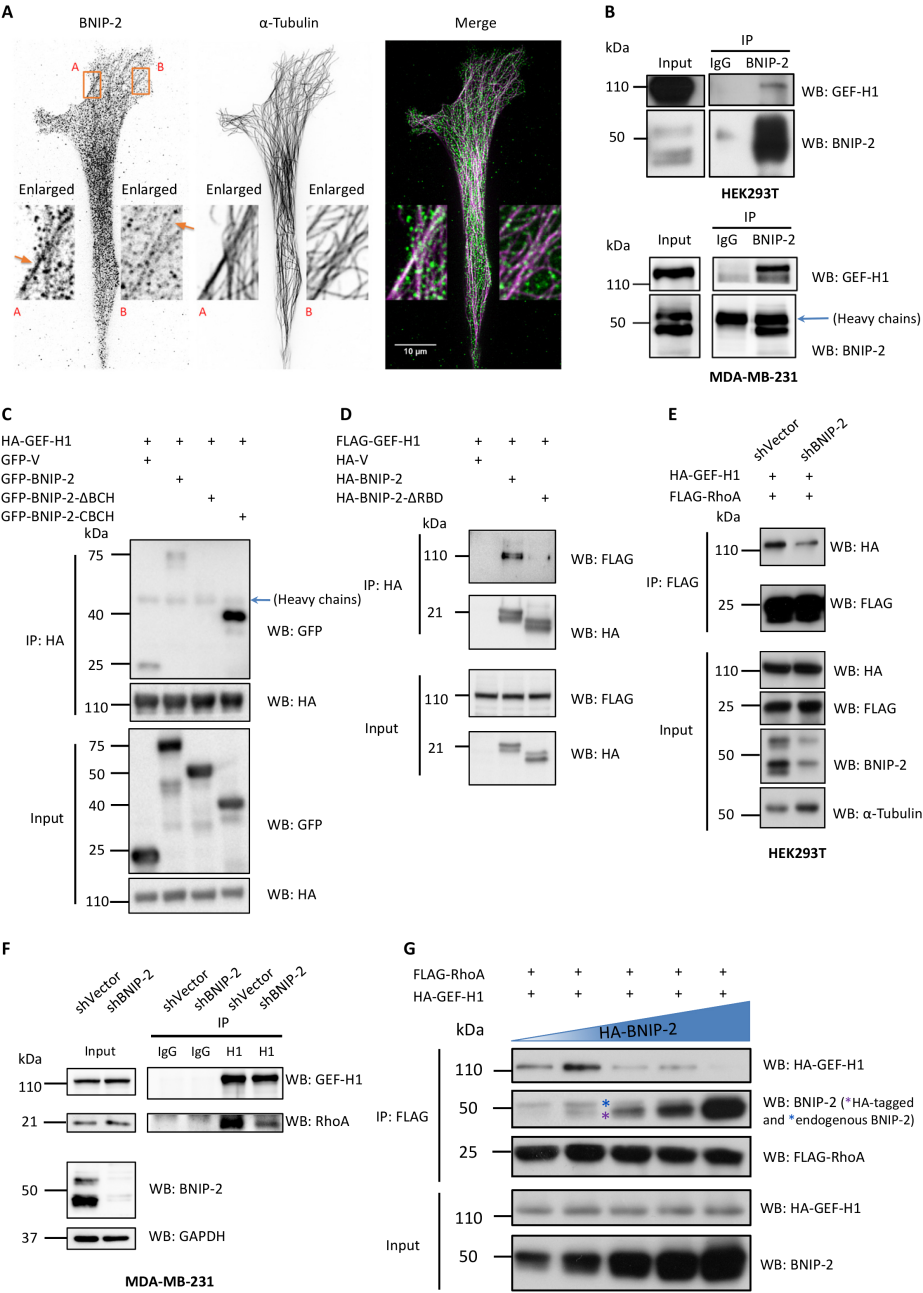
BNIP-2 binds GEF-H1 and scaffolds for RhoA/GEF-H1 signaling. (**A**) MDA-MB-231 cells stained for BNIP-2 and α-tubulin. Boxes A and B highlight the regions where BNIP-2 colocalizes with microtubules. (**B**) BNIP-2 interacts with GEF-H1 under physiological conditions. HEK293T (top) or MDA-MB-231 (bottom) cell lysates were incubated with BNIP-2 antibody or immunoglobulin G (IgG) control for immunoprecipitation and then Western blotted with GEF-H1 and BNIP-2 antibodies. The blue arrow denotes heavy chains. (**C**) BNIP-2 uses the C terminus to interact with GEF-H1. Lysates of HEK293T cells transiently expressing HA-GEF-H1 and GFP-vector, BNIP-2-FL, or different mutants were immunoprecipitated with anti-HA beads and then Western blotted with GFP or HA antibodies. The blue arrow denotes heavy chains. (**D**) BNIP-2 uses the RBD-like region in BCH domain to interact with GEF-H1. Lysates of HEK293T cells transiently expressing HA-tagged BNIP-2 or BNIP-2-ΔRBD with FLAG-tagged GEF-H1 were immunoprecipitated with anti-HA beads and then Western blotted with FLAG or HA antibodies. (**E**) Knockdown of BNIP-2 reduces interaction between RhoA and GEF-H1 in HEK293T cells. Lysates of shVector control and BNIP-2 knockdown HEK293T cells transiently expressing HA-GEF-H1 and FLAG-RhoA were immunoprecipitated with anti-FLAG beads and then Western blotted with FLAG, HA, BNIP-2, or α-tubulin antibodies. (**F**) Knockdown of BNIP-2 reduces interaction between RhoA and GEF-H1 in MDA-MB-231 cells. Lysates of control and knockdown MDA-MB-231 cells were incubated with GEF-H1 antibody or IgG control for immunoprecipitation and then Western blotted with GEF-H1, RhoA, BNIP-2, or GAPDH antibodies. (**G**) BNIP-2 has a scaffolding effect for RhoA and GEF-H1 interaction. Lysates of knockdown HEK293T cells transiently expressing HA-tagged GEF-H1, FLAG-tagged RhoA, and increasing amount of HA-BNIP-2 (illustrated by blue triangle) were immunoprecipitated with anti-FLAG beads and Western blotted with FLAG, HA, or BNIP-2 antibodies. The blue asterisk denotes the endogenous BNIP-2, and the purple asterisk denotes the HA-tagged BNIP-2.

We next investigated the molecular mechanism for BNIP-2 to activate RhoA. Previous work has shown that BNIP-2 decorates microtubules and traffics on microtubules (*27*). We verified the result by doing live imaging of BNIP-2 and the microtubule marker enconsin (movie S2) and immunostaining (Fig. 4A). GEF-H1 is a RhoA-specific RhoGEF that is microtubule associated. It is inactive when localizing on polymerized microtubules via its N terminus (*31*). Its localization to microtubules is also regulated via phosphorylation by kinases such as p21-activated kinase, extracellular signal–regulated kinase, microtubule affinity regulating kinase 2, and interaction with microtubule motor dynein light chain Tctex-1 (*32*–*34*). On the basis of the scaffolding feature of BCH domain and microtubule motor kinesin-1 binding ability of BNIP-2 (*27*), we investigated whether BNIP-2 scaffolds for RhoA and GEF-H1.

First, we conducted endogenous coimmunoprecipitation assay using cell lysates from human embryonic kidney (HEK) 293T or MDA-MB-231 cells and showed that BNIP-2 forms a physiological complex with GEF-H1 (Fig. 4B). We also showed that the C terminus of BNIP-2 that contains the BCH domain interacts with GEF-H1, whereas the Rho-binding region of BNIP-2 is also required to form a complex with GEF-H1 (Fig. 4, C and D).

Next, we studied whether BNIP-2 is required for RhoA/GEF-H1 interaction. HEK293T cell line with stable BNIP-2 knockdown was generated, and both control and stable BNIP-2 knockdown cells were transfected with FLAG-GEF-H1 and HA-RhoA for coimmunoprecipitation. Western blot analysis showed that knockdown of BNIP-2 reduced the interaction between RhoA and GEF-H1 (Fig. 4E). Similarly, we observed that MDA-MB-231 BNIP-2 knockdown cells have reduced interaction between RhoA and GEF-H1, as shown by endogenous coimmunoprecipitation (Fig. 4F). Furthermore, we tested whether BNIP-2 has a concentration-dependent scaffolding effect on the interaction between RhoA and GEF-H1 (Fig. 4G). In stable BNIP-2 knockdown HEK293T cells, a fixed amount of HA-GEF-H1 and FLAG-RhoA was transfected together with a different amount of HA-BNIP-2 for coimmunoprecipitation. We observed that increasing amount of BNIP-2 first enhanced the binding between RhoA and GEF-H1 (Fig. 4G, lane 2), and their binding decreased sharply in the presence of higher amount of BNIP-2 (Fig. 4G, lanes 3 to 5). These concentration-dependent effects of BNIP-2 on RhoA/GEF-H1 interaction, together with the consistent effects on RhoA activity (Fig. 2A), indicate that BNIP-2 functions as a scaffold for RhoA and GEF-H1. A model of the scaffold mechanism is shown in fig. S4.

From the TCGA-BRCA database, the RNA expression level of BNIP-2 is reduced by about 11% in Breast Invasive Carcinoma (TCGA-BRCA) tissues compared to normal tissues, while neither RhoA nor GEF-H1 has noticeable differences between cancer and normal samples. In contrast, BNIP-2 has a significant decrease in tumor samples (fig. S3A), which indicates that BNIP-2 is the limiting factor for breast cancer motility.

### BNIP-2 and its association with KLC-1 is required for GEF-H1–driven RhoA activation upon microtubule disassembly

**Fig. 5:**
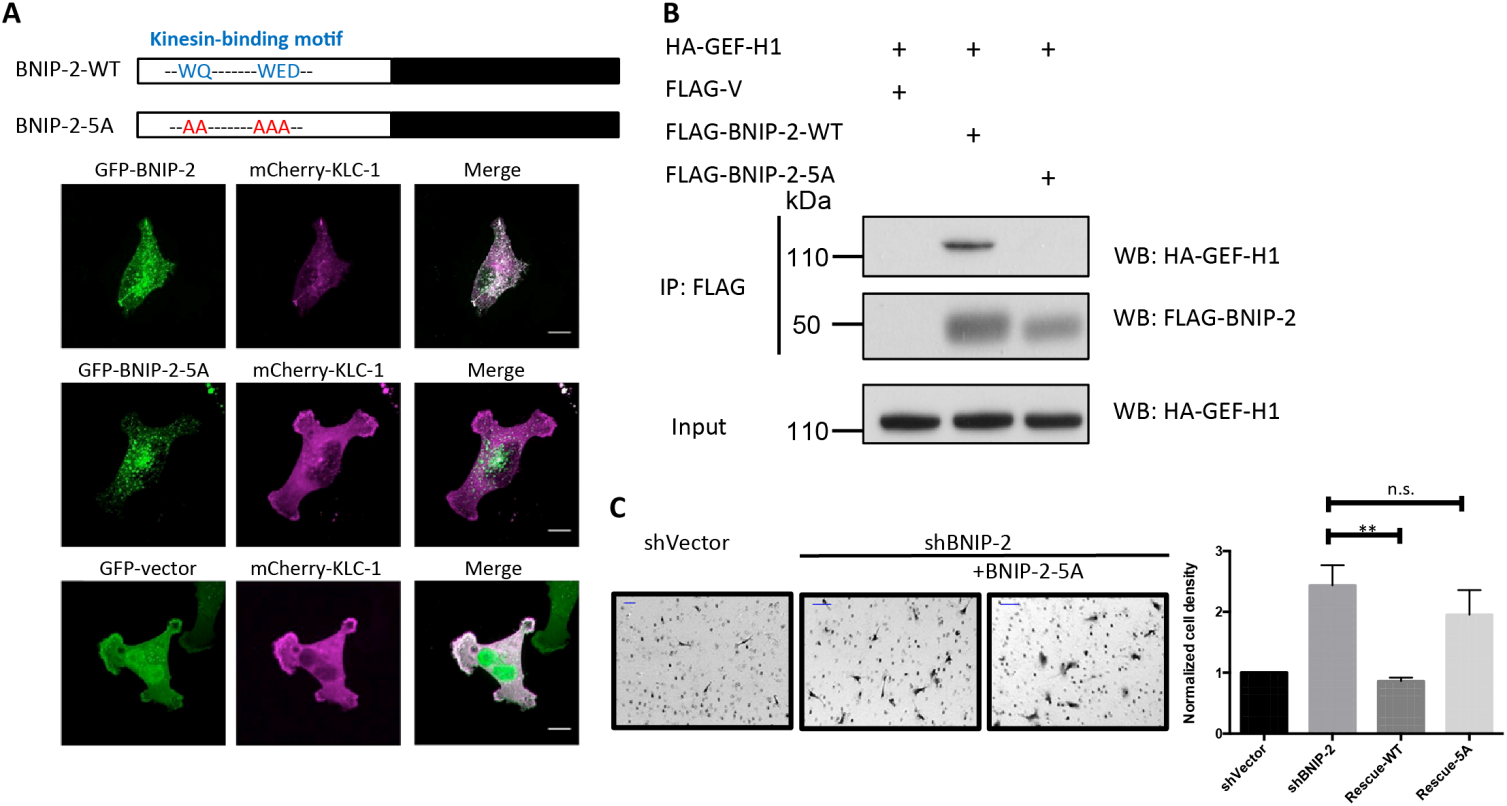
BNIP-2 association with KLC-1 is required for its GEF-H1 binding and migration control. (**A**) BNIP-2 associates with KLC-1. Schematic representation of BNIP-2-WT with the kinesin-binding motif and BNIP-2-5A mutant, which is unable to bind KLC-1, where WE-WED motif in the N terminus is mutated to five alanines. Bottom: MDA-MB-231 cells were transfected with mCherry-KLC-1 and GFP-BNIP-2-WT, GFP-BNIP-2-5A, or GFP-vector for live imaging. Maximum *Z*-projected images are presented. Scale bars, 10 μm. (**B**) KLC-1 binding motif of BNIP-2 is required for interaction with GEF-H1. Lysates of HEK293T cells transiently expressing FLAG-tagged BNIP-2-WT, BNIP-2-5A, or empty vector with HA-tagged GEF-H1 were immunoprecipitated with anti-FLAG beads and then Western blotted with FLAG or HA antibodies. FLAG-V stands for FLAG-vector. (**C**) BNIP-2-5A mutant cannot rescue cell motility increased by BNIP-2 knockdown. Representative images of transwell migration assay on MDA-MB-231 shVector control cells, shBNIP-2 cells, and BNIP-2 knockdown cells rescued with BNIP-2-5A mutant. Scale bars, 100 μm. Right: Statistical analysis for shVector control cells, shBNIP-2 cells, and BNIP-2 knockdown cells rescued with BNIP-2-WT or BNIP-2-5A mutant. Cell number per area was counted from randomly choosing sites and averaged. Final results presented here were normalized to the number of control cells (equals 1). Data are means ± SEM of three independent experiments, *P* < 0.01. n.s., not significant.

Because of the ability of BNIP-2 to traffic on microtubules (Fig. 4A) and to interact with the light chain of microtubule motor kinesin-1 (KLC-1) (*28*), we hypothesized that the binding of BNIP-2 to KLC-1 on microtubules may be important for its subsequent scaffolding effect for RhoA and GEF-H1. BNIP-2 mutant BNIP-5A, with substitution of KLC-1 binding motif WQ-WED to be five alanines, is reported to abolish KLC-1 binding (*28*). Confocal imaging showed that BNIP-2-WT (wild type) but not BNIP-2-5A colocalizes with KLC-1 (Fig. 5A), consistent with literature (*28*). Our coimmunoprecipitation results showed that mutation at BNIP-2-5A abolishes the binding to GEF-H1 compared to BNIP-2-WT (Fig. 5B). Introducing the increasing concentration of this mutant is unable to regulate RhoA activity in the same manner as BNIP-2-WT (fig. S5A). Furthermore, cell motility increased by BNIP-2 knockdown can no longer be fully rescued by the transfection of BNIP-2-5A mutant (Fig. 5C). These results strongly suggest that the interaction of BNIP-2 to kinesin-1 is important for the subsequent formation of BNIP-2/GEF-H1 complex, Rho activation, and cell migration.

**Fig. 6:**
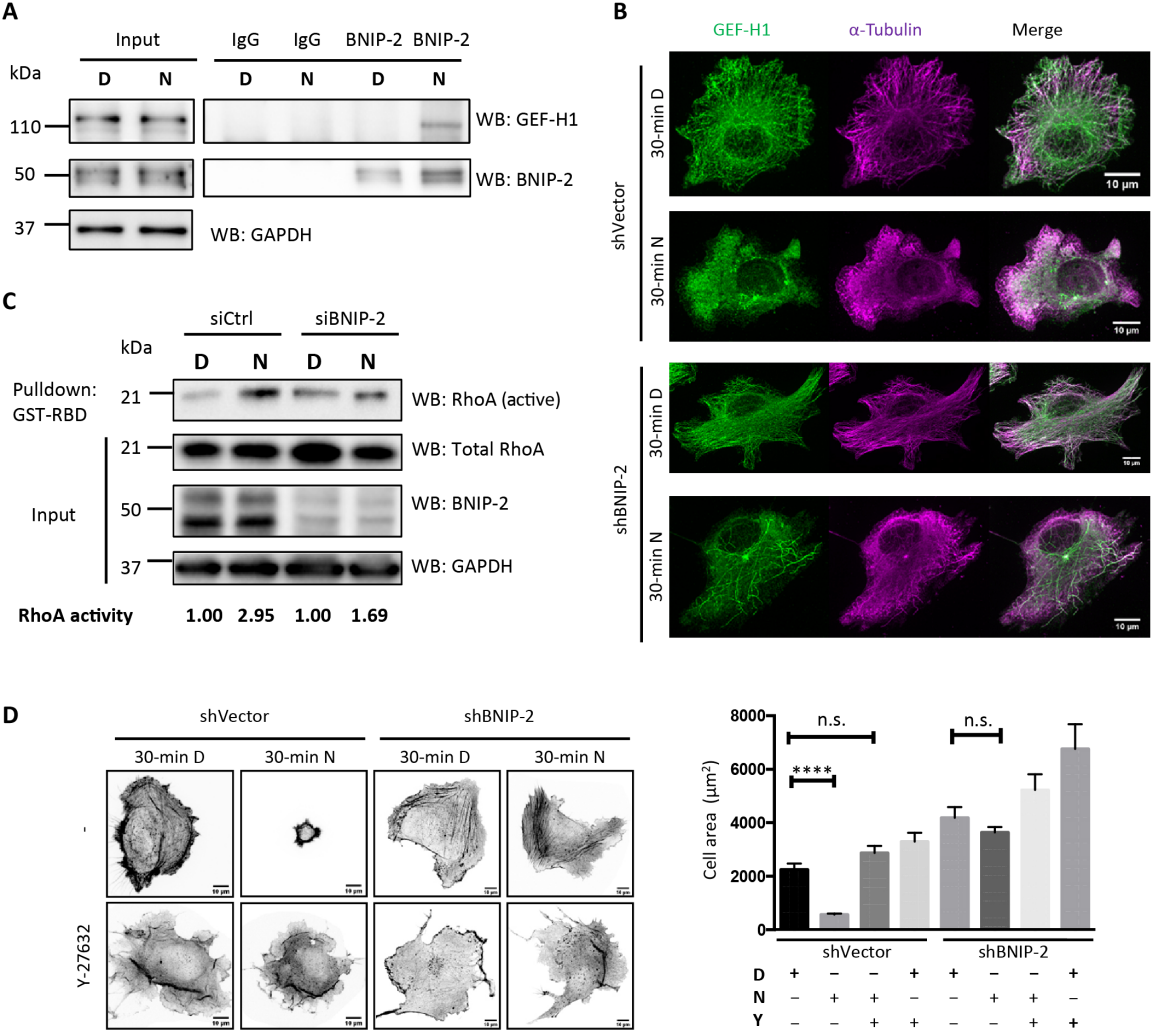
BNIP-2 is required for GEF-H1–driven RhoA activation upon microtubule disassembly. (**A**) Nocodazole treatment increases BNIP-2 and GEF-H1 interaction. MDA-MB-231 cells were treated with dimethyl sulfoxide (DMSO) (D) or 1 μM nocodazole (N) for 30 min and lysed. Lysates were incubated with BNIP-2 antibody or IgG control, incubated with Protein A/G PLUS-Agarose beads for immunoprecipitation, and then Western blotted with GEF-H1, BNIP-2, or GAPDH antibodies. (**B**) shVector control and BNIP-2 knockdown MDA-MB-231 cells were treated with DMSO or 1 μM nocodazole for 30 min, followed by immunostaining for α-tubulin and GEF-H1. Maximum *Z*-projected images are presented. (**C**) BNIP-2 is required for nocodazole-induced RhoA activation. MDA-MB-231 cells transfected with siControl and siBNIP-2 were treated with DMSO or 1 μM nocodazole for 30 min, lysed for RBD assay, and then Western blotted with RhoA, BNIP-2, or GAPDH antibodies. Quantification of Rho activity is displayed at the bottom with nocodazole normalized against DMSO for each cell line. (**D**) BNIP-2 and ROCK are required for nocodazole-induced cell rounding. shVector control and BNIP-2 knockdown MDA-MB-231 cells with or without 2-hour pretreatment with 20 μM Y-27632 (Y) were treated with DMSO or 1 μM nocodazole for 30 min and fixed for phalloidin stainning. Maximum *Z*-projected images are presented. Right: Statistical analysis for cell area. Data presented are means ± SEM, *P* < 0.0001. n.s., not significant.

As GEF-H1 is inactive when being sequestered by microtubules, we further investigated whether microtubules play a role in regulating the BNIP-2 scaffolding system. Upon microtubule depolymerization by nocodazole treatment, the interaction between BNIP-2 and GEF-H1 increases (Fig. 6A). It has been shown that nocodazole treatment releases GEF-H1 from microtubules and activates RhoA, and this activation is abolished when GEF-H1 is knocked down (*2*, *14*). We checked the subcellular localization of GEF-H1 and α-tubulin with or without nocodazole treatment. Many cells were rounded up after 30 min of nocodazole treatment, and immunofluorescence of those cells that remain attached revealed that the microtubules were disrupted and GEF-H1 was released from microtubules to cytoplasm (Fig. 6B). Since BNIP-2 displays stronger interaction with GEF-H1 upon microtubule depolymerization, we tested whether BNIP-2 plays a role in GEF-H1/RhoA activation upon microtubule disassembly. MDA-MB-231 cells showed activation of RhoA after nocodazole treatment (Fig. 6C, lanes 1 and 2). Knockdown of BNIP-2 showed much reduced activation of RhoA after nocodazole treatment (Fig. 6C, lanes 3 and 4). These results demonstrate that BNIP-2 is required for GEF-H1–mediated activation of RhoA upon microtubule disassembly. Furthermore, control cells treated with nocodazole significantly reduced their cell area because of cell rounding compared to cells with dimethyl sulfoxide (DMSO) treatment, while BNIP-2 knockdown cells showed no significant differences (Fig. 6D). In addition, pretreatment of the control cells with a ROCK inhibitor, Y-27632, was able to prevent the nocodazole-induced cell rounding (Fig. 6D), which strongly suggests that the cell rounding was due to the increased cell contractility downstream of RhoA activation by GEF-H1.

### BNIP-2 phenocopies GEF-H1 effects in microtubule disassembly–mediated cell rounding and focal adhesion dynamics

**Fig. 7:**
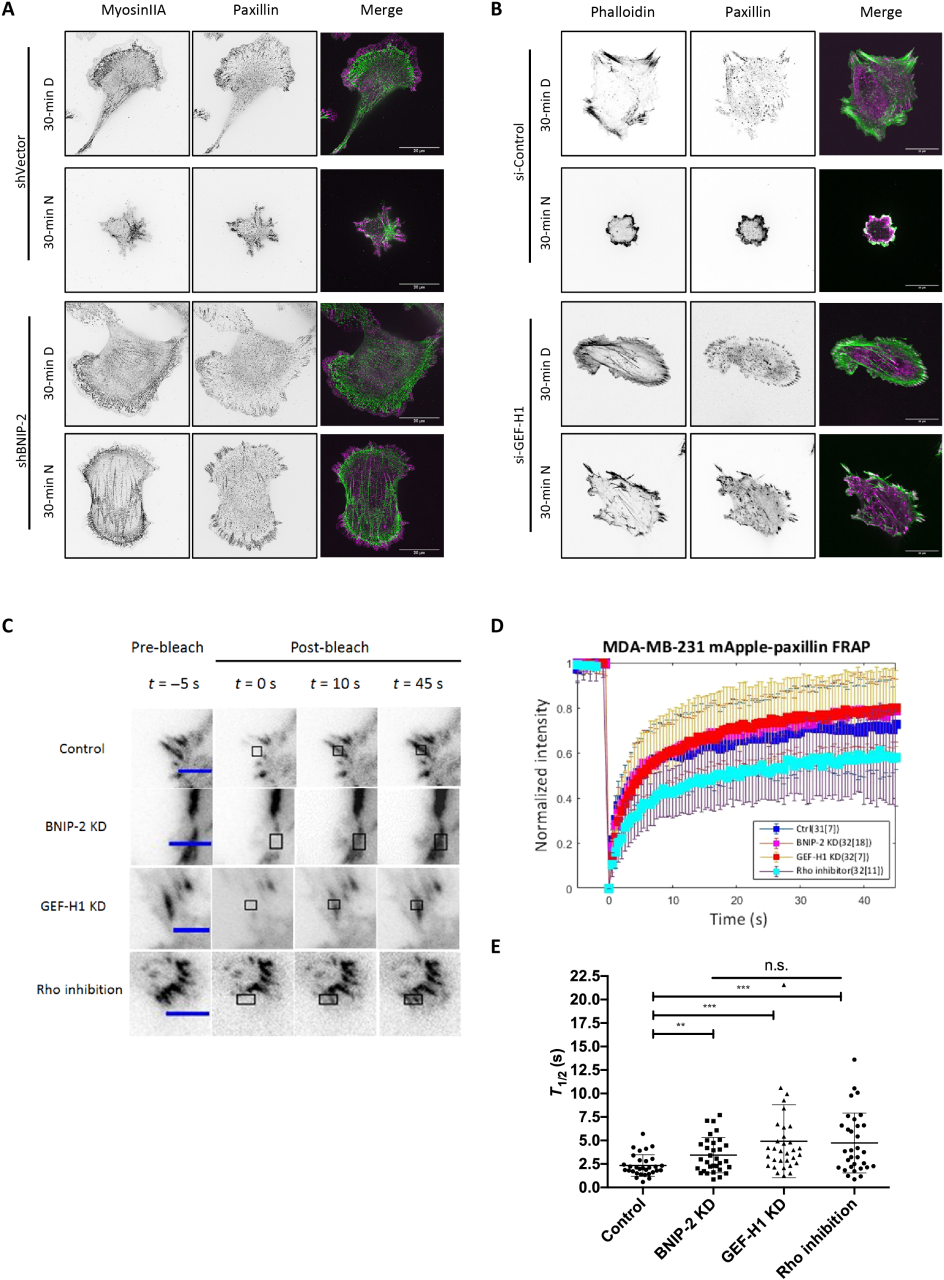
BNIP-2 phenocopies GEF-H1 effects in cell rounding upon nocodazole treatment and focal adhesion dynamics. (**A**) Nocodazole treatment causes dissolution of myosin filaments and focal adhesions. Top: shVector control MDA-MB-231 cells were treated with DMSO (D) or 1 μM nocodazole (N) for 30 min, followed by immunostaining for myosinIIA and paxillin. Bottom: BNIP-2 knockdown (KD) MDA-MB-231 cells were treated with DMSO or 1 μM nocodazole for 30 min, followed by immunostaining for myosinIIA and paxillin. Single-plane images are presented. Scale bars, 20 μm. (**B**) GEF-H1 knockdown cells have reduced cell rounding upon nocodazole treatment. Top: MDA-MB-231 cells transfected with control siRNA were treated with DMSO or 1 μM nocodazole for 30 min, followed by immunostaining for phalloidin and paxillin. Bottom: MDA-MB-231 cells transfected with GEF-H1 siRNA were treated with DMSO or 1 μM nocodazole for 30 min, followed by immunostaining for phalloidin and paxillin. Single-plane images are presented. Scale bars, 20 μm. (**C** to **E**) Focal adhesion turnover is affected by BNIP-2 knockdown, GEF-H1 knockdown, or Rho inhibition. (C) FRAP images of shVector control, BNIP-2 knockdown, GEF-H1 knockdown, and Rho-inhibited MDA-MB-231 cells expressing paxillin-mApple. Scale bars, 5 μm. (D) FRAP-relative fluorescence intensity curves of shVector control, BNIP-2 knockdown, GEF-H1 knockdown, and Rho-inhibited MDA-MB-231 cells expressing paxillin-mApple. The fluorescence intensity is normalized by the average prebleach intensity. (E) The mean ± SD half-life time (*T*_1/2_) of FRAP for each focal adhesion in shVector control, BNIP-2 knockdown, GEF-H1 knockdown, and Rho-inhibited MDA-MB-231 cells expressing paxillin-mApple. The half-life time is calculated from FRAP-relative fluorescence intensity (D). **P < 0.01 and ***P < 0.001.

In most reported cell lines such as HT1080 cells, microtubule disassembly induces GEF-H1 release and up-regulation of Rho activity, which results in increased focal adhesion size and myosin stacks (*15*). In MDA-MB-231 cells, the resulting effects from microtubule disassembly are cell rounding and disappearance of focal adhesions and myosin filaments (Fig. 7A, top). Cells with either knockdown of BNIP-2 (Figs. 6D and 7A, bottom) or knockdown of GEF-H1 (Fig. 7B, knockdown efficiency shown in fig. S6) showed similarly impaired cell rounding, as a symbol of resistance toward microtubule depolymerization. Since MDA-MB-231 cells do not show increased focal adhesion size upon microtubule disassembly, we examined the dynamics of focal adhesions under normal conditions to check whether BNIP-2 and GEF-H1 mediate RhoA and focal adhesions in the same direction. Fluorescence recovery after photobleaching (FRAP) experiments of the focal adhesion protein paxillin were carried out and showed slower turnover in knockdown of BNIP-2 and knockdown of GEF-H1 cells when compared to control cells (Fig. 7, C to E). Last, treatment with Rho inhibitor in control cells showed slower turnover of paxillin, similar to the impacts from the knockdown of BNIP-2 and knockdown of GEF-H1.

## DISCUSSION

### BNIP-2 as a scaffold for GEF-H1 and RhoA during cell migration

In this study, we have uncovered that BNIP-2 interacts with both RhoA and GEF-H1 (Figs. 1 and 4). BNIP-2 can either promote or inhibit RhoA activity depending on its expression level (Fig. 2A), consistent with the observation that the interaction between RhoA and GEF-H1 is also regulated by the relative expression level of BNIP-2 (Fig. 4, D to F). These results suggest that BNIP-2 is a scaffold protein for RhoA and GEF-H1. Scaffold proteins fine-tune RhoA activity on the basis of their concentration, which may result in different migratory behaviors between normal cells and cancer cells. For highly metastatic MDA-MB-231 cells with high Rho activity, enhanced RhoA activity suppresses migration (*3*, *35*). Our results showed that BNIP-2 knockdown reduces RhoA activity and increases MDA-MB-231 cell migration (Fig. 3, E, F, and I to J). It is consistent with our data from the TCGA-BRCA database that BNIP-2 is significantly down-regulated in tumor tissues compared to normal tissues (Fig. 3, A and B). Such correlation between BNIP-2 expression level and cell metastasis suggests that the expression level of BNIP-2 could be a marker for the cancer cell metastasis.

**Fig. 8:**
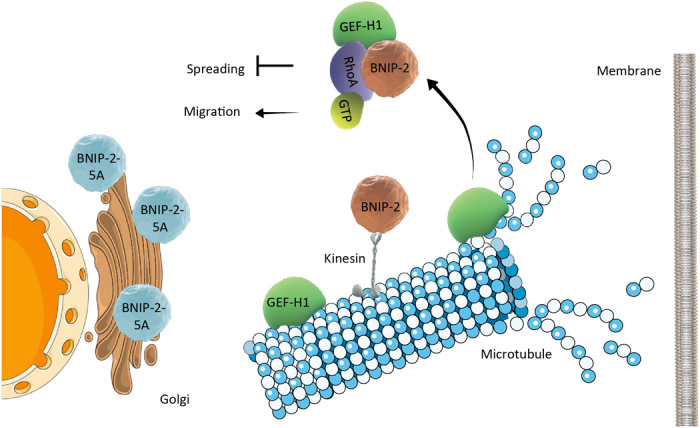
BNIP-2 as a scaffold for GEF-H1/RhoA activation in cancer cell migration. GEF-H1 plays an important role in the interplay between microtubules, actomyosin, and focal adhesions. After GEF-H1 is released from microtubules, it still requires BNIP-2 to act as a scaffold to activate RhoA via GEF-H1. When the levels of BNIP-2 are too low or too high, the interaction between GEF-H1 and RhoA and the levels of Rho–guanosine triphosphate are reduced. An optimal level of BNIP-2 promotes RhoA activation and inhibits cell migration. On the other hand, although microtubules sequester GEF-H1 from release, the binding of BNIP-2 to the microtubule motor kinesin-1 is required for the interaction between BNIP-2 and GEF-H1. This suggests that the BNIP-2/kinesin-1 trafficking pathway may regulate the dynamics of GEF-H1 on microtubules and release GEF-H1 in a spatially and temporally specific manner.

The scaffolding effect of BNIP-2 on GEF-H1/RhoA and cell migration is summarized in Fig. 8. Mechanistically, BNIP-2 interacts with both RhoA and GEF-H1 via its unique BCH domain using its Rho-binding region (RBD) (Figs. 1D and 4D). Since GEF-H1 needs to be in close proximity with RhoA to activate RhoA, the Rho-binding region (45 amino acids) of BNIP-2 may contain structurally distinct motifs for binding with RhoA and GEF-H1. It would be interesting to delineate how a scaffold domain like BCH domain could mediate the interaction between GEF-H1 and RhoA at the molecular level.

### BNIP-2 for microtubule-actomyosin interplay

Microtubules disassemble or disconnect from focal adhesions during cell migration, which releases GEF-H1 and promotes RhoA activation (*15*). We find that nocodazole-induced microtubule disassembly increases the interaction between BNIP-2 and GEF-H1 in MDA-MB-231 cells (Fig. 6A). When BNIP-2 is knocked down, GEF-H1 reduces the interaction with RhoA (Fig. 4, E and F) and thus unable to activate RhoA (Fig. 2A). This leads to reduced Rho activity, resulting in retarded cell polarization during cell spreading (Fig. 2, B and C) and enhanced single-cell three-dimensional migration and collective migration (Fig. 3). Furthermore, BNIP-2 knockdown shares several effects as GEF-H1 knockdown on the dynamics of focal adhesion under normal conditions as well as the RhoA activity and cell rounding phenotype upon treatment with nocodazole (Fig. 7). Our results suggest that even after GEF-H1 dissociates from microtubules, it still requires BNIP-2 to facilitate the activation of RhoA and complete the interplay between microtubules and actomyosin.

In this study, we observed some unique phenotypes in MDA-MB-231 cells. First, although many studies show that microtubule disassembly–induced Rho activation leads to increased focal adhesion size and enhanced myosin stacks, such activation causes cell rounding and dissolution of focal adhesions in MDA-MB-231 cells. The finding that cells pretreated with ROCK inhibitor abolished the cell rounding effect provides the evidence that cell rounding is due to high contractility (Fig. 6D). MDA-MB-231 cells could already have intrinsically high Rho activity and contractility. Further activation may have caused overwhelming cellular contraction so that they rapidly decouple from the substrate and dissemble myosin filaments (Fig. 7A). Second, although control cells show cell rounding when treated with nocodazole, BNIP-2 knockdown and GEF-H1 knockdown cells that retarded cell rounding show increased focal adhesion sizes (Fig. 7, A and B, bottom), suggesting that the effect of nocodazole on increasing focal adhesion size is evident in cells with lower contractility and could involve other Rho-activating mechanisms apart from GEF-H1. Last, although Rho can promote focal adhesion maturation, treatment with Rho inhibitor, BNIP-2-KD, or GEF-H1-KD all lead to reduced focal adhesion dynamics in MDA-MB-231 cells, indicating that focal adhesions are more stable upon RhoA inhibition. Hence, our observation of focal adhesion dynamics does not necessarily correlate with the migratory behavior of this cell line.

### BNIP-2 and KLC-1 could provide spatial regulation for GEF-H1/RhoA

Note that the BNIP-2-5A mutant that could not bind KLC-1 abolishes its interaction with GEF-H1, has no concentration-dependent scaffold effects on Rho activity, and fails to rescue the increased cell migration by BNIP-2 knockdown (Fig. 5, A to C and fig. S5). These findings indicate that BNIP-2 is not only a static scaffold for RhoA/GEF-H1, but it could also provide spatial regulation on RhoA/GEF-H1 via KLC-1–mediated trafficking on microtubules (Fig. 8). There are reports showing that RhoA can be trafficked on endocytic vesicles when stimulated by vascular endothelial growth factor (*36*) and GEF-H1 can interact with a Rab13-exocyst complex during cell migration (*32*, *37*), suggesting that KLC-1–mediated trafficking of BNIP-2 may have a role in RhoA/GEF-H1 (co)trafficking on microtubules. Furthermore, GEF-H1 can interact with dynein light chain (Tctex-1) so as to be sequestered and inactivated on microtubules (*38*). KLC-1 could have an opposite role in contrast to Tctex-1. Since KLC-1 localizes more like vesicles with the presence of BNIP-2 (Fig. 5A), BNIP-2 may function as an activating cargo for KLC-1 to traffic on microtubules and possibly to activate GEF-H1 by dissociating GEF-H1 from microtubules. Furthermore, more in-depth studies could be carried out to investigate where BNIP-2 scaffolds GEF-H1 and RhoA and whether BNIP-2 trafficking complex regulates microtubule stability. To address these questions, high-resolution live imaging and optogenetic tools are needed to investigate the localization and dynamics of BNIP-2, GEF-H1, and RhoA at the focal adhesion/microtubule interface under conditions such as microtubule depolymerization or trafficking.

To date, studies on how RhoA and GEF-H1 regulate cancer cell migration have shown different results in a cell line–dependent pattern (*12*, *13*, *39*, *40*). Our results suggest a complex regulation network of RhoA activity that is fine-tuned by microtubule-actomyosin interplay and a scaffold protein BNIP-2 in a concentration-dependent manner. It is therefore important that RhoA activity is examined not only on the spatiotemporal context but also on the nature and dynamic action of their specific scaffold(s). In this case, BNIP-2 links microtubule dynamics to RhoA activation and actomyosin contractility and focal adhesion dynamics to cell migration. Through better understanding of scaffold proteins such as BNIP-2 and their roles in the cross-talk between cytoskeleton and signaling, we envisage identifying novel cell migratory markers and pharmaceutical targets in different cancer types.

## MATERIALS AND METHODS

### Cell culture and transfection

MDA-MB-231 cells (American Type Culture Collection) were cultured in Dulbecco’s Modified Eagle’s Media (Hyclone) supplied with 10% fetal bovine serum (Gibco), 1 mM sodium pyruvate (Gibco), penicillin (100 U/ml), and streptomycin (100 mg/ml; Hyclone). Cells were transfected with Lipofectamine 2000 reagent (Invitrogen) or jetPRIME (Polyplus-transfection) according to the manufacturer’s instructions. HEK293T cells were cultured in RPMI 1640 medium (Hyclone) supplied with 10% fetal bovine serum, 10 mM Hepes, penicillin (100 U/ml), and streptomycin (100 mg/ml; Hyclone). Cells were transfected using Mirus (*Trans*IT-LT1) according to the manufacturer’s instructions. All cells were grown at 37°C with 5% CO_2_. For all cell lines, TrypLE Select Enzyme (Gibco) was used for passaging. Cells within passages 3 to 15 were used for all the experiments.

### Plasmids

Full-length BNIP-2 complementary DNAs (cDNAs) were amplified from human cDNA, reverse-transcribed from total RNA of Hela cells. The human RhoA, Rac1, and Cdc42 plasmids were gifts from the late A. Hall. The human GEF-H1 plasmid was a gift from Y. Nishimura (Bershadsky laboratory, Mechanobiology Institute, Singapore). The KLC-1 plasmid was created in the laboratory (*28*). All the cDNAs and mutants were subcloned in FLAG-, HA-, GFP-, and mCherry-tagged pXJ40 vectors (gift from E. Manser, Institute for Molecular and Cell Biology, Singapore). Glutathione *S*-transferase (GST)–rhotekin was provided by S. Schoenwaelder (Monash University, Australia). The paxillin-mApple plasmid was from the Davidson collection (Kanchanawong laboratory, MBI), and GFP-ensconsin was from the Bershadsky laboratory, Mechanobiology Institute, Singapore.

BNIP-2 plasmid template was used to generate BNIP-2 truncation and mutation constructs, including BNIP-2-CBCH, BNIP-2-ΔBCH, BNIP-2-ΔRBD, and BNIP-2-5A. BNIP-2 cDNA was ligated to pcDNA-FLAG vector for generation of stable overexpression cell lines. High-fidelity DNA polymerase PfuUltra (Stratagene) was used for site-directed mutagenesis. Constructs were sequenced to confirm sequence fidelity.

### Antibodies and chemicals

For Western blot, primary antibody catalog numbers and dilution factors are as follows: polyclonal anti–BNIP-2 antibodies were purchased from Sigma-Aldrich (HPA026843) and GeneTex (GTX114283) or self-purified from rabbit serum, as previously described (*28*); monoclonal anti–GEF-H1 (ab155785) was purchased from Abcam; monoclonal anti-RhoA was from Santa Cruz Biotechnology (sc-418); polyclonal anti–Phospho-Myosin Light Chain 2 (Ser^19^) antibody was from Cell Signaling Technology (#3671); monoclonal anti–α-tubulin was from Sigma-Aldrich (T9026); anti–glyceraldehyde-3-phosphate dehydrogenase (GAPDH) was from Life Technologies (Invitrogen); rabbit immunoglobulin G (IgG; sc-2027); and mouse IgG (sc-2025) were from Santa Cruz Biotechnology. The horseradish peroxidase secondary antibodies polyclonal antibody against FLAG and polyclonal antibody against HA were from Sigma-Aldrich. For immunostainings, all secondary antibodies conjugated with Alexa Fluor dyes and Alexa Fluor Phalloidin were from Life Technologies. For inhibitor treatment, Rho inhibitor I (catalog no. CT04) and Rho activator II (catalog no. CN03) were from Cytoskeleton Inc. DMSO, Y-27632, blebbistatin, and nocodazole were from Sigma-Aldrich.

### Knockdown experiments

ON-TARGETplus Human BNIP-2 siRNA-SMARTpool (L-011814-00-0005) and ON-TARGETplus Human GEF-H1 siRNA-SMARTpool (L-009883-00-0005) were purchased from Dharmacon. The efficiency of RNA interference (RNAi) was assayed by Western blotting to measure the endogenous protein level.

Knockdown by plasmid-based RNAi was carried out with the pGFP-V-RS vectors (OriGene) that expressed BNIP-2–targeting sequences. The BNIP-2–targeting sequence was designed with Ambion siRNA Designer and purchased from Sigma-Aldrich. Knockdown efficiency was determined by transient transfection of shRNA constructs and analyzing the cell lysates 48 hours after transfection by Western blotting. When compared with the empty vector control (shVector control), two shRNA constructs (shBNIP-2-1 and shBNIP-2-2) successfully depleted BNIP-2 by 70 to 80%. The sequences are shBNIP-2-1 (5′-GGAAGGTGTGGAACTTAAAGA-3′, targeting BCH domain in the C terminus) and shBNIP-2-2 (5′-GGAAGGTGTGGAACTTAAAGA-3′, targeting the first five amino acids in the N terminus). The shBNIP-2-2 construct was then used for subsequent experimentation for generation of stable knockdown cell lines.

### Generation of stable cell line

For generation of BNIP-2 knockdown cells, pGFP-V-RS-shBNIP-2-1 plasmid or empty vector control was transfected into MDA-MB-231 or HEK293T cells. Twenty-four hours after transfection, cell growth medium was changed to puromycin-containing medium. Cells were selected for single clones and tested for knockdown efficiency by Western blot analysis.

For generation of BNIP-2–overexpressing cells, pcDNA-FLAG-BNIP-2 plasmid or empty vector control was transfected into MDA-MB-231 cells. Twenty four hours after transfection, cell growth medium was changed to geneticin-containing medium. Cells were selected for single clones and tested for overexpression efficiency by Western blot analysis.

### Coimmunoprecipitation

Endogenous immunoprecipitation was conducted using Protein A/G PLUS-Agarose beads (sc-2003, Santa Cruz Biotechnology). Cells were lysed by ice-cold radioimmunoprecipitation assay (RIPA) buffer [50 mM tris (pH 7.3), 150 mM sodium chloride, 0.25 mM EDTA, 1% Triton X-100, 1% (w/v) sodium deoxycholate, and a mixture of protease inhibitors] and incubated with primary antibody or an equal amount of IgG and then Protein A/G PLUS-Agarose beads or Protein G magnetic beads. Exogenous immunoprecipitation was conducted using anti-FLAG M2 agarose beads (Sigma-Aldrich) for cells transfected with FLAG-tagged plasmids or anti-HA magnetic beads for cells transfected with HA-tagged plasmids, as indicated in each experiment. Cells were lysed in 300 μl of RIPA buffer at 24 hours after transfection, and immunoprecipitation was performed using M2 beads. In both cases, beads were extensively washed with lysis buffer three to five times and resuspended in 2× protein loading dye. Total input and immunoprecipitated samples were analyzed by Western blotting.

### RhoA activation assay

Assays for the active (guanosine triphosphate–bound) form of RhoA was performed, as described previously (*23*, *24*). Briefly, HEK293T or MDA-MB-231 cells were seeded on six-well plates. Cells were lysed in RBD buffer [50 mM tris-HCl (pH 7.4), 100 mM sodium chloride, 2 mM magnesium chloride, 1% Triton X-100, 10% glycerol, supplemented with 1 mM dithiothreitol and a mixture of protease inhibitors] and subjected to pulldown assays with GST fusion of the RBD of rhotekin, which would bind and detect active RhoA in vivo. Bound RhoA was separated on SDS–polyacrylamide gel electrophoresis gels and subjected to Western blot analysis with the RhoA antibody.

### Western blot analysis

The Western blot procedures were performed, as previously described (*28*). The results were visualized by either film developer or ChemiDoc Touch (Bio-Rad).

### Cell spreading assay

Cells passaged 1 day in advance were trypsinized and recovered in complete medium for 30 min. Subsequently, cells were seeded onto collagen-coated dishes and taken for time-lapse live-cell imaging using an Olympus IX81 inverted widefield microscope at 10× magnification.

### Immunofluorescence

For α-tubulin staining, cells were prefixed with 0.3% glutaraldehyde and 0.2% Triton X-100 in 1× phosphate-buffered saline (PBS) for 3 min at 37°C and then fixed by 4% paraformaldehyde (PFA) in PBS for 15 min at 37°C. After fixation, free aldehydes were quenched with sodium borohydride (5 mg/ml; Sigma-Aldrich) for 5 min. Samples were washed for 5 min in 1× PBS three times and incubated with blocking solution (2% bovine serum albumin and 0.2% Triton X-100 in 1× PBS) for 30 min. For GEF-H1 staining, cells were incubated with −20°C methanol for 10 min, permeabilized, and blocked with blocking solution for 60 min. For other stainings, cells were fixed with 4% PFA for 15 min at 37°C. Fixed cells were washed and permeabilized with blocking solution for 60 min. After fixation and blocking, samples were incubated with appropriately diluted primary antibodies in blocking solution for 60 min at room temperature, washed by 1× PBS three times, and incubated with Alexa Fluor–conjugated secondary antibodies. This was followed by three times 1× PBS washes and subsequent imaging.

### Search and summary of breast carcinoma datasets

Two sets of breast carcinoma microarray data analyzed in Fig. 3 (A and B) (GDS3853 and GDS3139) were found by searching in National Center for Biotechnology Information Gene Expression Omnibus DataSet Browser (www.ncbi.nlm.nih.gov/gds). TCGA-BRCA data plotted in fig. S3A were using the online Gene Expression Profiling Interactive Analysis (GEPIA) platform (http://gepia2.cancer-pku.cn/).

### Transwell migration assay

After cell trypsinization, cells were mixed with Trypan Blue Stain (Invitrogen) at 1:1 ratio. Cell number was counted using LUNA Automated Cell Counter. MB-231 cells (2 × 10^4^) in 200 μl of serum-free medium were placed on Falcon Permeable Support for a 24-well plate with 8.0-μm-pore Transparent Membrane (Corning, no. 353097) coated with collagen (Gibco) with the lower chamber filled with 700 μl of medium with 10% fetal bovine serum. After 6 hours, cells that did not penetrate the pores were removed by cotton swab. Migrated cells were fixed with 3.7% PFA in PBS, stained with 0.2% crystal violet solution for 1 hour, and imaged by an EVOS benchtop microscope or a Canon camera installed on an inverted Olympus microscope CKX41 at ×10 magnifications. Four random fields were chosen to image and quantify the cell number using ImageJ software (National Institutes of Health, Bethesda, MD).

### Collective migration assay

A commercial migration stencil insert (ibidi, no. 80209) was used to create a blocking space (500 μm wide) that separates two groups of confluent cells. Dishes were coated by collagen and air-dried before stencils were pressed onto the dish surface firmly. Cells were counted and seeded cell overnight to form collective group of cells. After removing the stencils, the cells were washed three times and placed into a live microscope. Cells were imaged by time-lapse live-cell imaging using an Olympus IX81 inverted widefield microscope at 10× magnification. Area closed was manually drawn and measured using ImageJ software.

### Confocal imaging

Confocal images were captured using a 100× Plan-Apo 1.45 numerical aperture (NA) oil immersion objective on a Nikon TiE microscope attached to a CSU-W1 spinning-disk confocal head. For images in Figs. 4A and 7A, Live-SR module (Gataca Systems) on the spinning-disk microscope was engaged during imaging and post processing of the images was carried out using Live-SR algorithm.

### Fluorescence recovery after photobleaching

FRAP experiments were performed with an iLAS2 module on the Olympus IX81 inverted microscope under Total Internal Reflection Fluorescence mode and with a 100× UApoN 1.49 NA oil immersion objective. FRAP experiments were conducted on live cells expressing paxillin-mApple 24 hours after seeding on 27-mm glass bottom. Cells were maintained at 37°C and 5% CO_2_ in a stage-top chamber. The bleach settings for FRAP images acquisition were set up as 5 s before bleach at 1-s intervals, 300 ms for photobleaching, and 45 s after bleach at 500-ms intervals.

### Quantitative analysis of FRAP

Regions of interest (ROIs) were manually drawn around unbleached area of focal adhesions for control, empty area for background, and bleached area of focal adhesions for measurements with ImageJ software. The area and integrated intensity of each ROI were acquired in ImageJ, and photobleach/background corrections were further performed with the formula

$$I_{\text{FRAP}}=(I_{\text{FRAP}}-\frac{I_{\text{background}}}{A_{\text{background}}}\times A_{\text{FRAP}})/(\frac{I_{\text{unbleach}}}{A_{\text{unbleach}}}-\frac{I_{\text{background}}}{A_{\text{background}}})$$

where *I*_FRAP_ is the integrated intensity of each FRAP ROI, *I*_background_ is the integrated intensity of background ROI for each image, *A*_background_ is the area of background ROI for each image, *A*_FRAP_ is the area of each FRAP ROI, *I*_unbleach_ is the integrated intensity of bleach correction ROI for each FRAP image, and *A*_unbleach_ is the area of bleach correction ROI for each FRAP image.

Data were normalized relative to the average prebleach fluorescence intensity. The half-life time of FAs was the result of FRAP exponential recovery fitting, performed with the following equation

$$I=A(1-e^{-\frac{t}{\tau }})$$

$$T_{1/2}=\text{ln}(2)\times \tau$$

where *I* is the normalized intensity during the FRAP recovery, *A* is the normalized end-value of recovered intensity, which is normalized relative to the average prebleach fluorescence intensity, *t* is the time after bleaching, τ is mean life time of recovery, and *T*_1/2_ is the half-life time of recovery.

### Image analysis

Phase-contrast data were used for quantifying cell shape changes during cell spreading. In-house Fiji macro was used for segmenting the cells across time. For data with very low contrast, segmentation was performed manually. The area and aspect ratio of segmented cells were then measured using Analyze Particles. For fluorescent data, the cell area was measured using Analyze Particles after applying Gaussian smoothing and thresholding. Counting cell number in transwell assay and measurement of gap closure area in the collective migration assay were conducted using ImageJ software.

### Statistical analysis

Experimental data are presented as means ± SEM. All statistical analyses were performed using analysis of a two-tailed Student’s *t* test with GraphPad Prism 6 (GraphPad Software). Differences were considered statistically significant if the *P* value is less than 0.05 (*P < 0.05, **P < 0.01, ***P < 0.001, and ****P < 0.0001).

## Supplementary Material

aaz1534_Movie_S2.avi

aaz1534_Movie_S1.avi

aaz1534_SM.pdf

## SUPPLEMENTARY MATERIALS

Supplementary material for this article is available at http://advances.sciencemag.org/cgi/content/full/6/31/eaaz1534/DC1

## Appendix

View/request a protocol for this paper from *Bio-protocol*.
