# Supplementary material for: BNIP-2 retards breast cancer cell migration by coupling microtubule-mediated GEF-H1 and RhoA activation
Source: Sci Adv. 2020 Jul 31;6(31):eaaz1534. doi: 10.1126/sciadv.aaz1534 (PMC7399486; doi:10.1126/sciadv.aaz1534)
Supplement: aaz1534_SM.pdf [file aaz1534_SM.pdf]

Science Advances
AAAS

advances.sciencemag.org/cgi/content/full/6/31/eaaz1534/DC1

Supplementary Materials for

BNIP-2 retards breast cancer cell migration by coupling microtubule-mediated GEF-H1 and RhoA activation

Meng Pan, Ti Weng Chew, Darren Chen Pei Wong, Jingwei Xiao, Hui Ting Ong, Jasmine Fei Li Chin, Boon Chuan Low*

*Corresponding author. Email: dbslowbc@nus.edu.sg

Published 31 July 2020, *Sci. Adv.* **6**, eaaz1534 (2020)
DOI: 10.1126/sciadv.aaz1534

The PDF file includes:

Figs. S1 to S6
Legends for movies S1 and S2

Other Supplementary Material for this manuscript includes the following:

(available at advances.sciencemag.org/cgi/content/full/6/31/eaaz1534/DC1)

Movies S1 and S2

Supplementary Figures

Figure S1

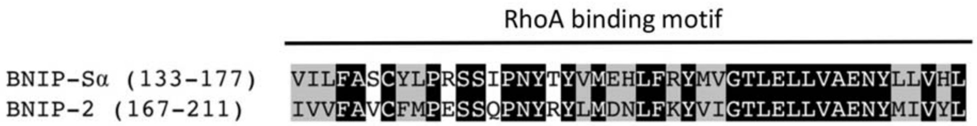

Figure S1. Identification of putative RhoA binding motif (167-211) in BNIP-2. Alignment was performed between the known RhoA binding motif reported in BNIP-Sα *(22)* and the BCH domain of BNIP-2. The RhoA binding motifs in both BNIP-2 and BNIP-Sα shared high homology.

Figure S2

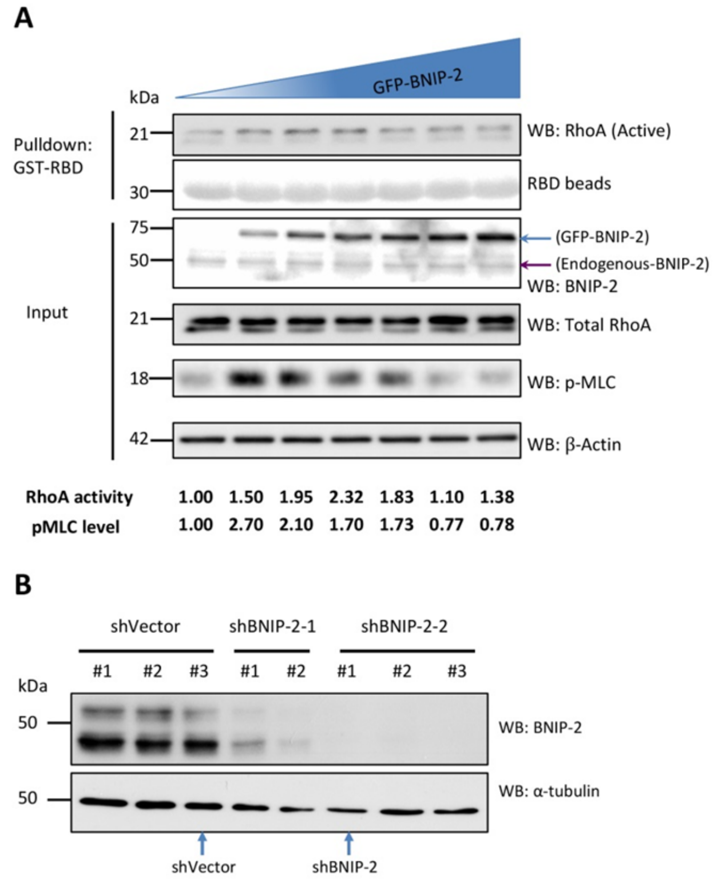

Figure S2. BNIP-2 scaffolds for RhoA activity and protein expression level of BNIP-2 knockdown stable cells. (A) A representative repeat for the result shown in Fig. 2A with

wider range of BNIP-2 at low concentrations. Lysates of MDA-MB-231 cells transiently transfected with gradually-increasing amount of GFP-BNIP-2 (illustrated by blue triangle) were used for pulldown with GST-RBD and then Western blotted with RhoA, BNIP-2, pMLC, and β-actin antibodies. The ratio of active RhoA to total RhoA and the ratio of pMLC to β-actin are normalized to lane 1 and labeled at the bottom. (B) Protein expression level of BNIP-2 knockdown stable cell lines. Cells were lysed and probed with BNIP-2 and α-tubulin antibodies. Different subclones of shVector, shBNIP-2-1 and shBNIP-2-2 were screened. The control and BNIP-2 knockdown cell lines used in this study are highlighted by blue arrows. Note that BNIP-2 has five bands representing five isoforms, all of which are reduced after BNIP-2 knockdown.

Figure S3

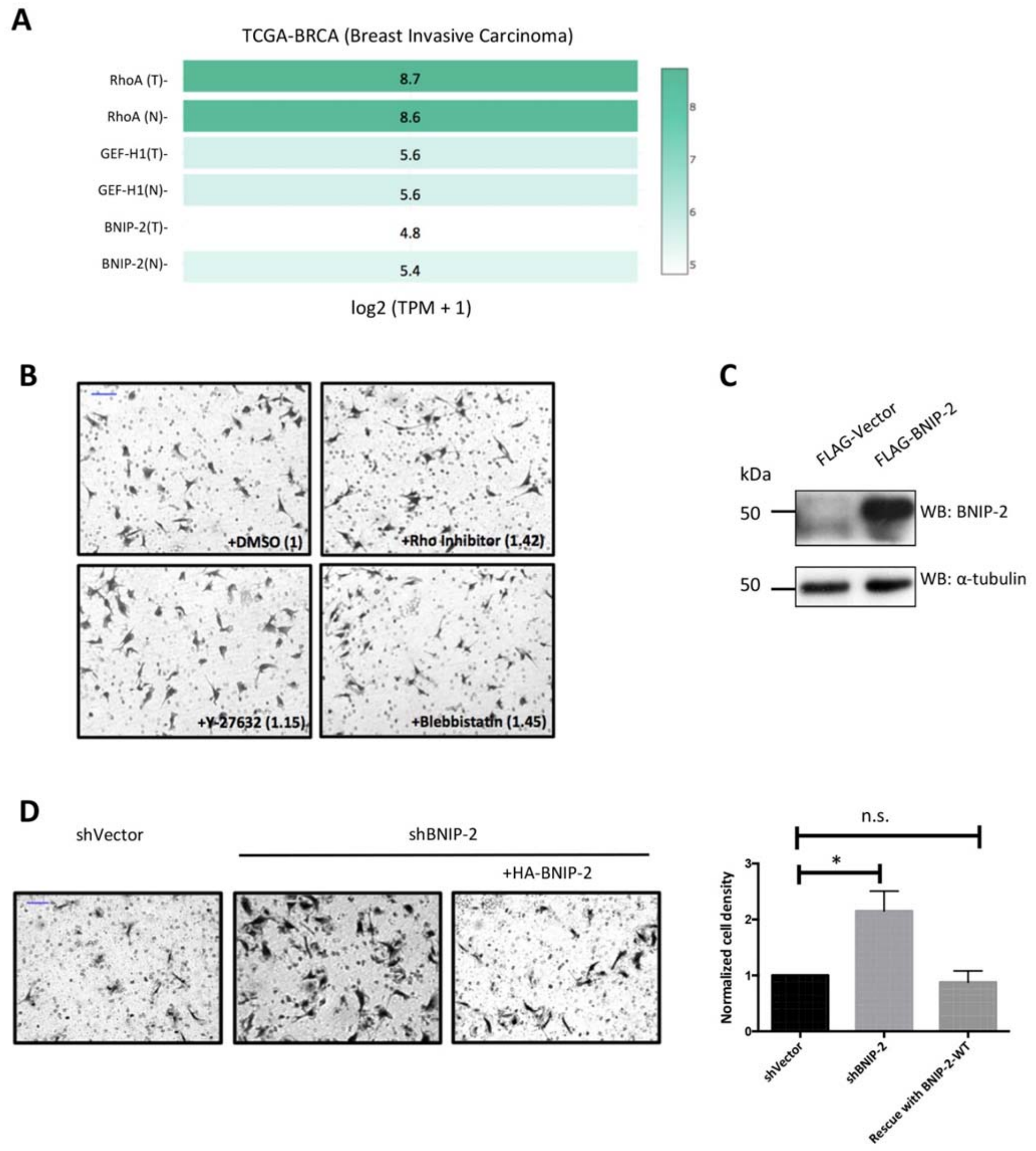

Figure S3. BNIP-2 downregulation and Rho inhibition promote breast cancer cell migration. (A) BNIP-2 is down-regulated in breast cancer. The RNA expression level of BNIP-2 is reduced for about 11% in Breast Invasive Carcinoma (TCGA-BRCA) tissues compared to normal tissues, while the RNA expression levels of RhoA and GEF-H1 have negligible differences. This plot indicates that BNIP-2 is the limiting factor for breast cancer motility. Values in the center of each bar: log2 (TPM + 1). Data was plotted using the online GEPIA platform (http://gepia2.cancer-pku.cn/). BRCA: Breast invasive carcinoma; T: Tumor (from TCGA tumor samples, n = 1085); N: Normal (n=291, from TCGA normal samples (n=112) and GTEx normal samples (n=179)). GEPIA: Gene Expression Profiling Interactive Analysis. (B) Rho Rho/ROCK/myosin inhibition increases cell migration. Representative images of transwell migration assay on MDA-MB-231 control cells treated with DMSO, Rho inhibitor (C3 transferase), ROCK inhibitor (Y-27632), and myosin inhibitor (Blebbistatin). Cell number per area was counted from four randomly choosing four sites and averaged. Final results shown in brackets were normalized to the number of cells with DMSO treatment (equals 1). Scale bar, 100 μm. (C) Protein expression level of BNIP-2 overexpressing stable cell lines. Control and BNIP-2 overexpressing cells were lysed and probed with BNIP-2 and α-tubulin antibodies. (D) Cell motility increased by stable BNIP-2 knockdown is rescued by BNIP-2. Representative images of transwell migration assay in shVector cells, shBNIP-2 cells, and knockdown cells with transfection of HA-BNIP-2. Scale bar, 100 μm. (Right) Statistical summary for this experiment by plotting cell numbers counted from four random fields from each group normalized to the average cell number of shVector cells. Final results presented here were normalized to the average number of control cells (equals 1). Data are means ± SEM of four independent fields. (*, $P<0.05$).

Figure S4

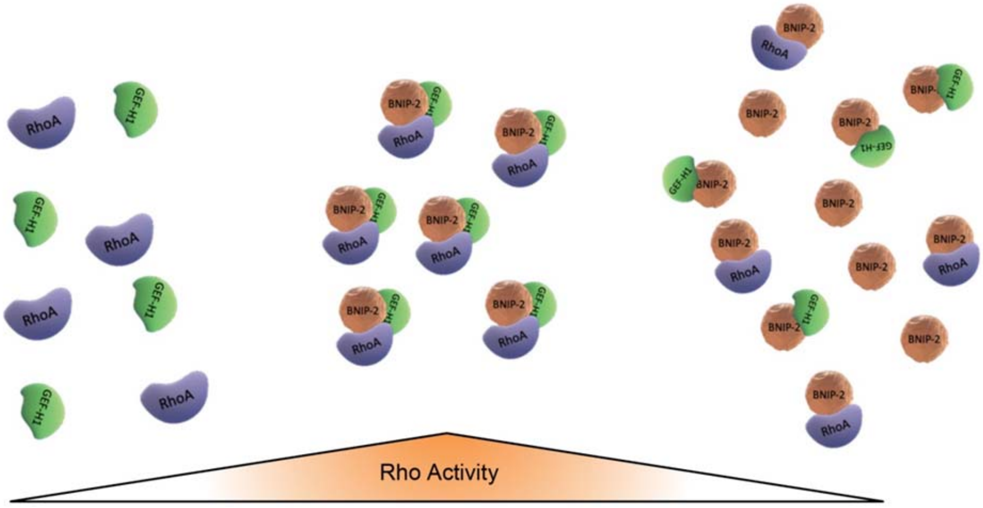

Figure S4. Illustration of scaffold tethering and titration effects of BNIP-2 on RhoA and GEF-H1. BNIP-2 tethers signaling partners RhoA and GEF-H1 to closer proximity for

interaction. Increasing BNIP-2 concentration can promote the interaction betwenn RhoA and GEF-H1 (middle) until that exceeds the concentrations of RhoA and GEF-H1,when BNIP-2 titrates RhoA and GEF-H1 into separate complexes and inhibit their interaction (right). According to this model, overexpression of BNIP-2 can exert either stimulatory or inhibitory effects on Rho activity depending on its concentration relative to RhoA and GEF-H1.

Figure S5

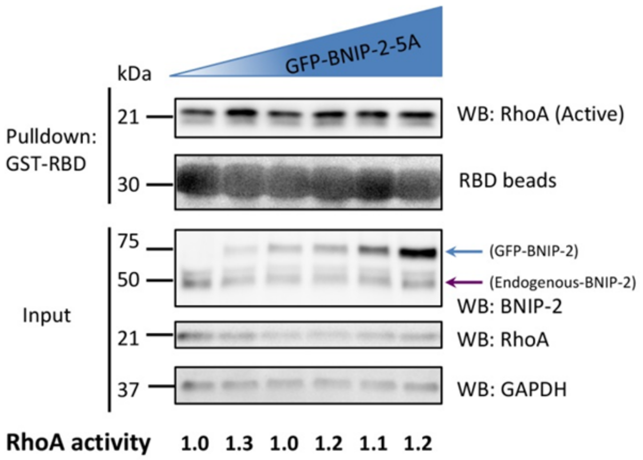

Figure S5. BNIP-2-5A mutant abolishes the scaffolding effect on RhoA activity. Lysates of MDA-MB-231 cells transiently transfected with gradually-increasing amount of GFP-BNIP-2-5A (illustrated by blue triangle) were used for pulldown with GST-RBD and then Western blotted with RhoA, BNIP-2, and GAPDH antibodies. The ratio of active RhoA to total RhoA is normalized to lane 1 and labeled at the bottom.

Figure S6

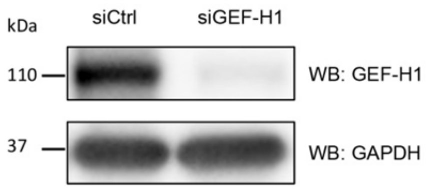

Figure S6. Knockdown efficiency of GEF-H1 siRNA. Cells with control siRNA or GEF-H1 siRNA were lysed and probed with GEF-H1 and GAPDH antibodies.

Supplementary Movies

Supplementary Movie 1

Control (left) and BNIP-2 knockdown (right) MDA-MB-231 cells were imaged just after cell seeding on collagen-coated plates. Phase-Contrast images were taken using Olympus EZ live microscope. The segmented cell boundary for aspect ratio analysis is shown in the blue outline. The frames were recorded at 2-minute intervals over a period of 54 minutes. The screenshots from this movie is shown in Figure 2B. Scale bar, 20 μm.

Supplementary Movie 2

BNIP-2 traffics on microtubules. MDA-MB-231 cells transfected with GFP-ensconsin and mCherry-BNIP-2 were imaged using W1 spinning disk microscope. The frames were recorded at 1-minute intervals over a period of 40 minutes. The similar experiment but using immunostaining of BNIP-2 and α-tubulin and also showing BNIP-2 co-localizes with microtubules is shown in Figure 4A.
